# Subconjunctival Mass as Rare Presentation of Even Rarer Intraocular Medulloepithelioma: A Case Report

**DOI:** 10.31729/jnma.5269

**Published:** 2021-01-31

**Authors:** Santosh Chaudhary, Vinayak Regmi, Sangeeta Shah, Purbesh Adhikari

**Affiliations:** 1 Department of Ophthalmology, B.P. Koirala Institute of Health Sciences, Dharan-18, Sunsari, Nepal; 2 Department of Pathology, B.P. Koirala Institute of Health Sciences, Dharan-18, Sunsari, Nepal

**Keywords:** *case report*, *enucleation*, *medulloepithelioma*

## Abstract

Medulloepithelioma is a rare childhood embryonal tumor arising from the non-pigmented ciliary epithelium of the pars plicata. We report a case of an 11-year-old male who presented with painless loss of vision of the right eye for the last three years and progressively increasing mass on the superior aspect of the globe for the last three months. On ocular examination, a firm, non-tender mass of 4cm × 3cm was noted in the superior aspect of the globe. CT-Scan of the orbit was suggestive of a foreign body with a haemorrhage or infection. The patient underwent enucleation with minimal manipulation. Histopathological examination of the enucleated globe revealed medulloepithelioma.

The intraocular medulloepithelioma presentation is often late and masquerading, which may lead to extraocular extension and metastasis and ultimately unfavorable prognosis.

## INTRODUCTION

Medulloepithelioma is a rare childhood intraocular tumor.^1^ It usually develops from the non-pigmented ciliary epithelium of the pars-plicata, but sometimes it may also arise from the iris, retina, and optic nerve head.^2–4^

Though rare, medulloepitheliomas are the second most common type of pediatric primary intraocular tumors.^5^ They have clinical characteristics similar to retinoblastoma and are challenging to differentiate from them clinically.^6^ Small medulloepitheliomas can go undetected. Extraocular extension in medulloepithelioma occurs in about 7-18% of cases, which has an unfavorable prognosis.^7,8^

Here, we would like to report a case of intraocular medulloepithelioma presented to us as a subconjunctival mass.

## CASE REPORT

**Figure 1:**
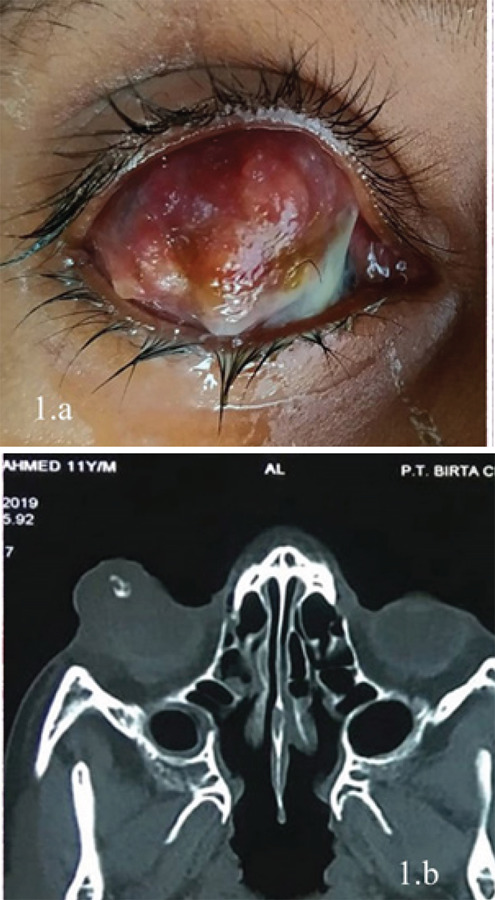
a. Clinical photograph demonstrating subconjunctival mass. b. CT-Scan image of orbit demonstrating hyperdense focus with surrounding thickening and irregular wall of the globe.

An 11-year-old male presented with a painless mass over the superior aspect of the globe for the last three months which was progressively increasing in size. The child had progressive, painless loss of vision in the same eye for the last three years. On ocular examination, visual acuity was no perception of light in the same eye and 6/6 in the other. Firm, non-tender mass measuring 4cm x 3cm was noted in the superior aspect of the globe (Figure 1.a). It was covered by conjunctiva in the posterior half, while conjunctiva was adherent on the anterior half. Its surface was irregular with some vascularization. The cornea was hazy due to scarring, and inner details could not be evaluated. The examination of the left eye was within the normal limit. Systemic examination was normal with no palpable lymph nodes. B-scan ultrasound showed homogenous opacity in the anterior part of the eye with moderate reflectivity persisting up to 70dB. CT-Scan of the orbit revealed hyperdense focus attached to the anterior surface of the eyeball with surrounding thickening, likely foreign body with an irregular wall of the globe with areas of internal hyperdensity likely a hemorrhage or infection (Figure 1.b).

After consent, enucleation was performed with minimal manipulation under general anesthesia with the orbital implant and conformer. The specimen was sent for histopathological examination.

**Figure 2:**
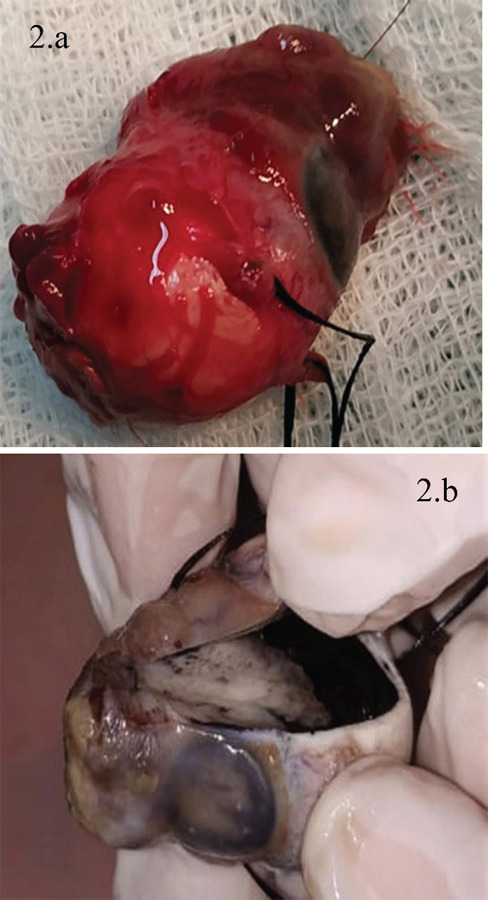
a. Enucleated Globe. b. Gross Section.

**Figure 3:**
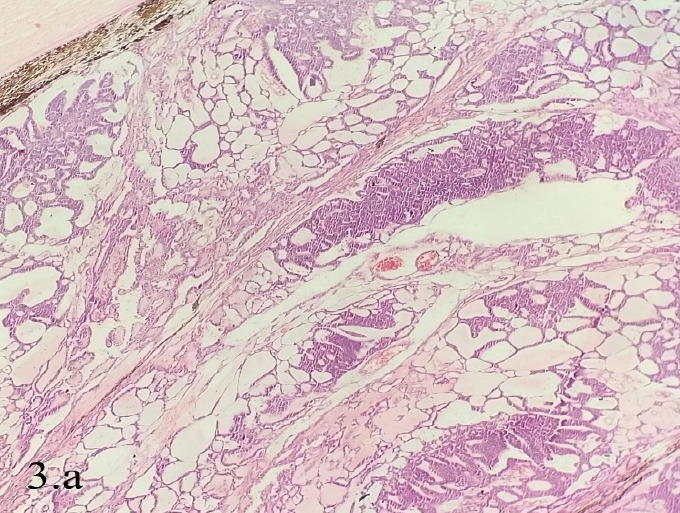

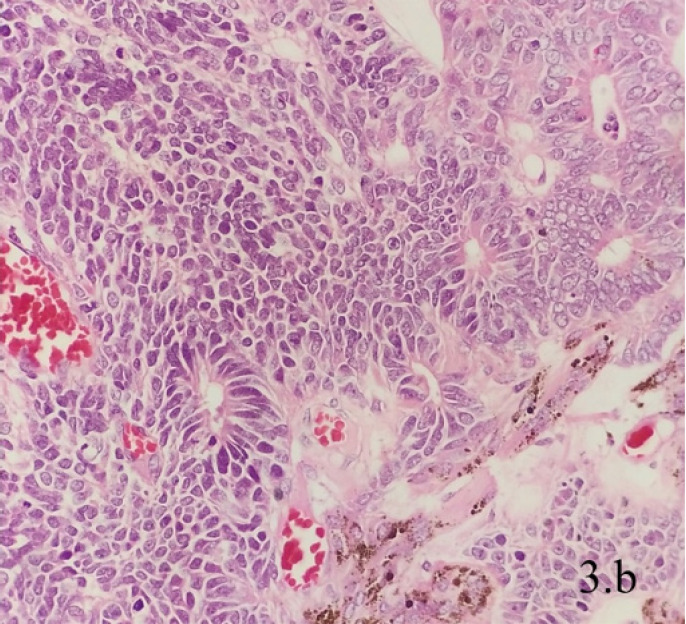
Histopathological Examination. a. Thick bands of polarized epithelium resembling medullary epithelium along with thinner cellular strands in a pool of acellular myxoid stroma (Hematoxylin and Eosin stain, 100x). b. Areas of poorly differentiated focus showing several Flexner-Wintersteiner rosettes are present (Hematoxylin and Eosin stain, 400x).

Gross examination of a cross-section of the enucleated globe revealed a solid grey lesion with exophytic and endophytic components (Figure 2.a and b). Histopathological examination revealed thick bands of polarized epithelium resembling medullary epithelium along with thinner cellular strands of acellular myxoid stroma (Figure 3.a). The tumor cells aggregated radially along the central space forming Flexner Wintersteiner rosette (Figure 3.b). It also involved the anterior chamber and vitreous cavity, but the optic nerve stump was free of tumor. This was highly suggestive of medulloepithelioma grade II. The origin of medulloepithelioma could not be confirmed.

The child is under regular follow-up. He is healthy, and there is no recurrence seen at one year of follow-up.

## DISCUSSION

Medulloepithelioma is a rare intraocular tumor presenting in children. It has varying and delayed presentation making it a challenge to diagnose and manage the tumor properly.

Brought and Zimmerman in 1978 studied 56 patients, of which 10 (18%) had invasion through the cornea or sclera or optic nerve. All 10 cases underwent enucleation, of which one case underwent additional orbital radiation and systemic chemotherapy and another patient underwent exenteration. Four cases lost their lives, and 3 cases lost to follow-up. Rest three were doing well two and nine years after therapy.^8^ Kaliki et al., in 2012, in the analysis of 41 cases of ciliary body medulloepithelioma, 7% had an extra scleral extension on presentation. Two cases underwent enucleation, and one underwent exenteration. All three cases later had metastasis. The average duration of metastasis to diagnosis was nine months.^7^

A tumor that is confined to the globe has an excellent prognosis, with a 5-years survival rate of 90-95% after enucleation. But an extension of tumor to extra scleral tissue dramatically increases metastasis and disease recurrence with an overall poor prognosis.^9^

Though there has been a lack of standard treatment of extraocular medulloepithelioma in literature because of the rarity of the disease, chemotherapy has been used with variable prognosis.^9^

Our patient underwent enucleation with minimal manipulation technique without touching the tumor. Despite the extra scleral extension, the child has not shown any signs of recurrence or metastasis over one year.

To conclude, subconjunctival mass as a presentation of intraocular medulloepithelioma is rare, and diagnosis is often delayed, making it a challenge to manage appropriately. When confined to the globe, it has a favorable prognosis. Unfortunately, delay in diagnosis may lead to extra scleral extension and an unfavorable prognosis.

## References

[1] Cardell BS, Starbuck MJ (1959). Diktyoma. Br J Ophthalmol.

[2] Vadmal M, Kahn E, Finger P, Teichberg S (1996). Nonteratoid Medulloepithelioma of the Retina with Electron Microscopic and Immunohistochemical Characterization. Pediatr Pathol Lab Med.

[3] Takei H, Florez L, Moroz K, Bhattacharjee MB (2007). Medulloepithelioma: Two unusual locations. Pathol Int.

[4] Morris T, Garner A (1975). Medulloepithelioma involving the iris. Br J Ophthalmol.

[5] Saunders T, Margo CE (2012). Intraocular medulloepithelioma. Arch Pathol Lab Med.

[6] Lee J, Choung HK, Kim YA, Kim N, Khwarg SI (2019). Intraocular medulloepithelioma in children: Clinicopathologic features itself hardly differentiate it from retinoblastoma. International Journal of Ophthalmology.

[7] Kaliki S, Shields CL, Eagle RC, Vemuganti GK, Almeida A, Manjandavida FP (2013). Ciliary body medulloepithelioma: Analysis of 41 cases. Ophthalmology.

[8] Broughton WL, Zimmerman LE (1978). A clinicopathologic study of 56 cases of intraocular medulloepitheliomas. Am J Ophthalmol.

[9] Tadepalli S, Shields CL, Shields J, Honavar S (2019). Intraocular medulloepithelioma - A review of clinical features, DICER 1 mutation, and management. Indian Journal of Ophthalmology.

